# Meniscus Regeneration With Multipotent Stromal Cell Therapies

**DOI:** 10.3389/fbioe.2022.796408

**Published:** 2022-02-09

**Authors:** Yun-Feng Zhou, Di Zhang, Wan-Ting Yan, Kai Lian, Zheng-Zheng Zhang

**Affiliations:** ^1^ Department of Orthopedics, Xiangyang No.1 People’s Hospital, Hubei University of Medicine, Xiangyang, China; ^2^ Department of Orthopedics, Sun Yat-sen Memorial Hospital, Sun Yat-sen University, Guangzhou, China; ^3^ Department of Obstetrics-Gynecology, Xiangyang No.1 People’s Hospital, Hubei University of Medicine, Xiangyang, China; ^4^ School of Medicine, Sun Yat-sen University, Shenzhen, China

**Keywords:** mesenchymal stem cell, meniscus repair, regenerative medicine, tissue engineering, biotherapy

## Abstract

Meniscus is a semilunar wedge-shaped structure with fibrocartilaginous tissue, which plays an essential role in preventing the deterioration and degeneration of articular cartilage. Lesions or degenerations of it can lead to the change of biomechanical properties in the joints, which ultimately accelerate the degeneration of articular cartilage. Even with the manual intervention, lesions in the avascular region are difficult to be healed. Recent development in regenerative medicine of multipotent stromal cells (MSCs) has been investigated for the significant therapeutic potential in the repair of meniscal injuries. In this review, we provide a summary of the sources of MSCs involved in repairing and regenerative techniques, as well as the discussion of the avenues to utilizing these cells in MSC therapies. Finally, current progress on biomaterial implants was reviewed.

## 1 Introduction

Located between the femoral condyle and tibia plateau cartilage in the knee joint, meniscus is a crescent-shaped fibrocartilaginous tissue. This semilunar disk decreases the stress of the tibiofemoral joint by increasing the congruency and the contact area of it (Englund et al., 2012). It also serves as a shock absorber and secondary stabilizer with a possible role in joint lubrication and proprioception (Makris et al., 2011). It has been reported that the meniscus is highly heterogeneous in cellular and extracellular matrix composition, as well as biomechanical properties (Morejon et al., 2020). The structural integrity and biological function would be impaired due to meniscus lesion, which ultimately lead to the deterioration of the joint and accelerate the development of osteoarthritis for the excessive concentrated forces adding on the articular cartilage (Danso et al., 2017).

Due to the differences in its blood supply and cell composition, the internal tissue composition of the healed meniscus was changed and could not be restored to the original state, especially in its relatively hypocellular and hypovascular inner edge. Currently, the prevailing theory for the treatment of meniscus-related lesions is to preserve its integrity as much as possible. Repair of the meniscal tear with a suture is a commonly used method (Chen et al., 2018; Zhang et al., 2020). However, the reported failure rates ranged from 0 to 38% for inside-out repairs and reached ∼80% for all-inside suture techniques (Barber-Westin and Noyes, 2014; Zhang et al., 2020). For patients with a large area of meniscus deficiency or widespread degeneration, meniscal allograft could be transplanted to maintain the function. It can effectively enhance objective knee stability and insignificantly narrow the joint space with a 5-year follow-up (Saltzman et al., 2017). However, this operation often requires the removal of the whole meniscus, including the remaining normal tissue. Drilling of extra bone tunnels is also needed for fixation, which is a more complex procedure with small incisions. Furthermore, the donor-matching process and the procurement of the graft before the surgery should also be taken into consideration. The transplantation of live cells in the allograft also carries the risks of disease transmission and the activation of immune response (Kurzweil et al., 2018165). In recent years, the newly developing regenerative medicine techniques have provided new hope for the treatment of damaged tissues. Regenerative medicine with MSCs has been receiving a growing attention in meniscal repair for their abilities of self-renew and multilineage differentiation (Pabbruwe et al., 2010; Embree et al., 2016).

With highly proliferative capacity, MSCs have been proven to be reliable cell sources for meniscus repair in clinical and preclinical studies. These cells differentiate into mature cells in the targeted tissue and generate an extracellular matrix so as to reconnect the damaged region or form new tissue, and display a similar morphology and function with adjacent tissue (Cossu et al., 2018). Several materials have been studied for their meniscal regenerative efficacy, whether absorbable or non-absorbable, natural, or synthetic (Zhang et al., 2015). Two kinds of techniques have been developed in meniscus regenerative medicine: cell-based and cell-free. While the former attempts to use scaffolds seeded with MSCs, the latter tends to implant scaffold into the joint without cells, repairing the meniscus by recruiting endogenous MSCs (Guo et al., 2018). In general, meniscus regeneration strategies using various MSC sources have been well documented in the literature, which can possibly provide a new strategy for meniscus repair (Twomey-Kozak and Jayasuriya, 2020; Dai et al., 2021). Nevertheless, a huge challenge still lies in mimicking its natural anisotropic structure. According to the published researches, the repair of meniscus defect is mostly regenerated with a homogeneous structure, which could not completely restore its function (Zhang et al., 2019; Li et al., 2021).

In this review, we generalized the sources of MSCs and descripted their biochemical characteristics and general situation. We also give a brief introduction to the MSCs utilizing approaches in meniscus regeneration. Both cell-based and cell-free strategies as well as one- or two-step methods will be involved in our discussion.

## 2 MSC Sources

**FIGURE 1:**
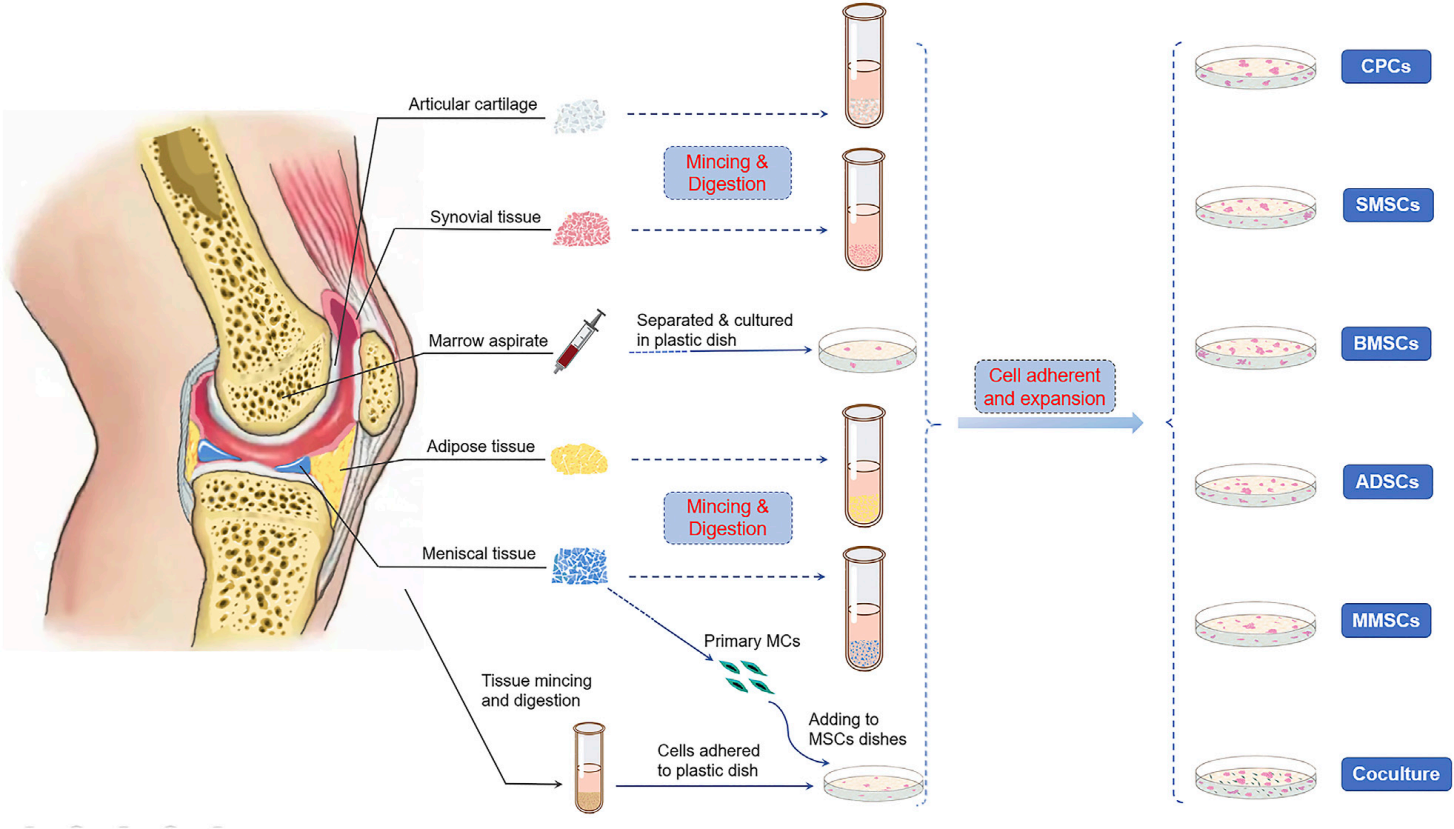
Cell sources of multipotent stromal cells (MSCs) in meniscus repair and regeneration. Bone marrow, synovium, cartilage, adipose tissue, and the meniscus itself were reported to be able to isolate stem/progenitor cells. Primary meniscus cells (MCs) also cocultured with MSCs from other tissues to induce directional differentiation.

MSCs could be obtained from various musculoskeletal tissues, such as bone marrow, synovium, adipose, cartilage, vessel, tendon, muscle, and meniscus itself. The International Society for Cellular Therapy (ISCT) put forward the criteria to define human MSCs (Dominici et al., 2006): expressing CD105, CD73, and CD90, and lack expression of CD45, CD34, CD14 or CD11b, CD79α or CD19, and HLA-DR surface molecules; besides, it must be plastic-adherent when maintained in a standard culture condition and preserve the ability of trilineage differentiation when incubated in an induced medium. In this section, we will focus on the application of various MSCs and their repair/regeneration efficacy for meniscus regeneration (Figure 1).

### 2.1 Bone Marrow Stem Cell

BMSCs are one of the most extensively studied and the most commonly explored source for tissue regeneration. Surface markers CD271, CD146, CD90 (Thy1), CD106 (VCAM1), CD105, CD51 (integrin α5), and CD140α (PDGFRα, also known as PDGF receptor α) have been shown to express its molecular detection and identification (Isern et al., 2013; Mabuchi et al., 2013). It is worth mentioning that the latter two markers were only detected in fetal BMSCs (Pinho et al., 2013). The key mechanism for the successful repair using BMSCs is the formation of ECM and its connection with surrounding normal tissues in the defect zone. The trophic factors secreted by these cells, which possibly can recruit resident cells, also contribute to this process (Caplan and Dennis, 2006). However, several drawbacks hinder the application of BMSCs. First, with less than 0.02% of bone marrow cells being BMSCs, the quantity is relatively limited (Alvarez-Viejo et al., 2013). Second, BMSCs are prone to develop into hypertrophic cells in cellular culture or tissue regenerative studies (Bilgen et al., 2018). Third, the operation of cell harvesting increases the risk of pain-suffering, infection, and morbidity of the donor site (Ma et al., 2018). What is more, the regenerative ability of BMSCs is largely dependent on the age of the donor: cells derived from the elder usually show weaker proliferation and differentiation capabilities.

### 2.2 Synovium-Derived Mesenchymal Stem Cell

In the early 21st century, SMSC was first isolated from the synovial membrane surrounding the joints. Due to its outstanding chondrogenic differentiation ability, it has received growing attention in recent years (Horie et al., 2012b; Nakagawa et al., 2015; Kondo et al., 2017). Compared with BMSCs, the SMSCs show greater colony-forming ability. It is reported that in SMSCs, 1 colony could be formed in 12.5–80 nucleated cells compared with 1 in 10^3^–10^4^ in BMSCs (Jo et al., 2007). SMSCs express CD105, CD73, CD140b, SSEA3, CD271, CD90, CD44, and CD34 (Matsumura et al., 2017). CD271 is a marker related to the differentiation potential, and the number of CD271-positive SMSCs increased in the expansion culture *in vitro* (Del Rey et al., 2016). Besides, CD90 was also highly expressed in SMSCs, which might be strongly correlated with the great chondrogenic potential of SMSCs retained over four passages (Sakaguchi et al., 2005; Roche et al., 2009). Meanwhile, the production of osteocalcin and alkaline phosphatase (ALP) in the SMSC group was nearly 10-fold lower than that of the BMSC group (Roche et al., 2009), which partially explains the finding that BMSCs are more prone to osteogenesis, while SMSCs incline to chondrogenesis. Treatment with SMSC transplantation also showed a protective effect on articular cartilage in aged cynomolgus macaque models (Kondo et al., 2017). Additionally, unlike BMSCs, the proliferation rate of SMSCs could be maintained regardless of age, and this superior character provides exciting news for tissue regeneration.

### 2.3 Adipose-Derived Mesenchymal Stem Cell

Fibroblast-like cells originated from processed lipoaspirate were reported to be expanded cultivated easily, expanded efficiently *in vitro*. These cells also exhibited adipogenic, osteogenic, chondrogenic, and myogenic differentiation potential when cultured in a medium containing lineage-specific differentiative factors (Zuk et al., 2001). It was reported that the number of stem cells per milliliter of lipoaspirate is about 8-fold higher than that of the bone marrow. 2% of nucleated cells are stem cells in lipoaspirate, while only 0.001–0.004% of them are stem cells in the bone marrow (Strem et al., 2005). Debnath’s research revealed that genetic stability of the exponentially growing human ADSCs was maintained without any clonal alterations until passage 5 (Debnath and Chelluri, 2019). By flow cytometry screening, these cells were found expressed CD29, CD44, CD71, CD90, CD105/SH2, and SH3, and absent for CD31, CD34, and CD45 (Zuk et al., 2002). Migration of these cells was confirmed in rabbit models, in which ADSCs were labeled with superparamagnetic iron oxide (SPIO) and were defected in the defect of meniscus (Qi et al., 2016). Compared with the control group, both the gross and histological findings suggested that allogenic ADSC transplantation facilitated the repair of the defected area (Toratani et al., 2017a). These research works demonstrated that ADSCs could effectively migrate to the meniscal defect zone and play an important role in meniscus repair.

### 2.4 Cartilage-Derived Chondrogenic Progenitor Cell

Several studies (Dowthwaite et al., 2004; Hiraoka et al., 2006) reported a chondrocyte subpopulation with progenitor-like characteristics. Among these cells, Notch-1 played a vital role in the process of colony-forming and multipotential differentiation. It has been reported that molecular markers, such as CD29, CD49, CD90, CD44, CD151, and CD166, have been detected in CPCs (Grogan et al., 2007; Candela et al., 2014), and the frequency of cells with molecular marker CD105+ and CD106+ was also increased in OA cartilage, while it was decreased in normal human cartilage (Alsalameh et al., 2004). Additionally, higher chondrogenic gene expression of chondrogenic genes was found in CPCs than in BMSCs, suggesting that these cells have a natural advantage in inducing chondrogenesis (Xue et al., 2015). Currently, most studies in the application of CPCs have focused on cartilage regeneration, and few concentrate on meniscus repair. According to Jayasuriya et al. (Jayasuriya et al., 2019), different single cell lines were successfully generated by CPCs, which confirmed the function of CPCs in mediating the rebinding and remodeling of torn meniscal tissues. The well-reintegrated torn meniscus explant demonstrated their proliferative and reparative capacity. The study also shows that these cells were better than BMSCs at preventing terminal differentiation and hypertrophy (Buma et al., 2004). Although the healing potential of CPCs and their superiority have been reported, more detailed research is still needed to verify their efficacy in meniscal regeneration.

### 2.5 Meniscus-Derived Mesenchymal Stem Cell

Unlike the MSCs previously mentioned, this type of stem cell has only been discovered and reported in recent years. The MMSC was used for intra-articular injection to treat meniscal tears in the past few years (Shen et al., 2013; Shen et al., 2014). The meniscus tissues were collected, digested, and cultured until a colony was formed *in vitro*. The authors found these cells with several markers, including CD44, CD90, CD106 and CD105.whereas were negative for CD45 and CD34. (Gamer et al., 2017). What is more, these cells showed better colony-forming capacity than BMSCs and SMSCs, which could be a unique characteristic and superiority of MMSCs for meniscus regeneration. Single-cell RNA sequencing (sc-RNA seq) has been proven to be a powerful technique in investigating the characteristics and the intra-relationship of cell subtypes from certain tissue. By using sc-RNA seq, Sun et al. (Sun et al., 2020) identified seven cell clusters in the healthy human meniscus, among which endothelial cells (EC) and fibrochondrocyte progenitors (FCP) might be meniscus progenitors by the following pseudotime analysis. This was further confirmed by their expression of MSC marker MCAM (CD146) as well as colony-forming and multidifferentiation capacity *in vitro*. However, Ding (Ding and Huang, 2015) found that colonies formed by MMSCs were fewer in number and smaller in size than those formed by BMSCs. They also reported that stem cells isolated from rabbit meniscus share similar properties with BMSCs, for no significant differences were found in gene markers and immunostaining results. Most of MMSCs were originated from “Red Zone,” the vascular region with good healing potential (Seol et al., 2017). In addition, a larger number of cells expressing CD34 were detected in the peripheral region than inner zone by immunohistochemical staining and flow cytometry (Osawa et al., 2013). Therefore, the authors speculated that the higher multilineage differentiation potential should be attributed to CD34 expression. These findings may help to explain the better histological healing in the peripheral region. Besides, it has been pointed out that whether the repair process occurred spontaneously was uncertain, and growth factors and chemotactic agents might play an important role in MMSCs’ migration and differentiation (Seol et al., 2017). For this reason, we assume that exogenous bioactive substances might be conductive to the homing of endogenous MMSCs after injury.

### 2.6 MSCs and Meniscus Cell Coculture

It was reported that coculturing with differentiated cells could direct MSCs’ differentiation (Tan et al., 2010). Matthies et al. (Matthies et al., 2013) found that when human BMSCs were cocultured with MCs, the formation of ECM was significantly enhanced *ex vivo*. According to Kremer et al. (Kremer et al., 2017), the products of coculturing equine BMSCs and MCs were much better than those that under the monoculture condition, and similar to native meniscus with respect to phenotype and composition. It was reported that the 50:50 (BMSC:MC) generated maximal GAG retention and showed optimal mechanical performance (McCorry et al., 2016). But Cui reported that the 25%: 75% ratio leads to the highest level of chondrogenic production and lowest hypertrophic gene expression (Cui et al., 2012). In another study (Xie et al., 2018), the coculture system of SMSCs/MCs at the ratio of 3:1 showed higher proliferation and more sGAG secretion than the monoculture did. Compared to SMSCs seeded on the construct separately, coculture with MCs yielded a greater cell survival rate and higher expression of chondrogenic markers (Tan et al., 2010). The effect of ADSCs cocultured with primary human MCs was also explored in a 3D porous scaffold (Weiss et al., 2017), and the mRNA expression level of ACAN in the coculture group was much higher than that in the ADSC group or MC group.

To sum up, MSCs from various tissues showed positive results regarding the enhancement of meniscal repair under 2D or 3D culture, *in vitro* or *in vivo*. BMSCs were the most thoroughly studied cell types in meniscal regenerative researches, followed by SMSCs and ADSCs; relatively fewer studies have been counted on CPCs and MMSCs. Various studies have confirmed their effect in promoting meniscal healing. The limited quantity and tendency to hypertrophy might be the primary obstacles. Coculture could reduce hypertrophy and promote the directed differentiation into fibrochondrocytes, which might provide a valid approach to address the drawbacks of a limited number of MCs and its tendency to hypertrophy during differentiation of MSCs. However, no consensus has been reached on the optimal ratio for co-cultivation. In general, MSCs from different sources share some similarities, while they also maintain their own unique characteristics. For example, SMSCs held superiority in colony formation and anti-hypertrophy, ADSCs had the advantage in cell quantity, and CPCs in cartilage-directed differentiation. These characteristics could provide guidance for researchers to choose which seeded cells to be used in meniscal repair studies. Taking their verified regenerative capabilities into consideration, it is reasonable for us to believe that the source of MSCs would not be the most limiting factor affecting meniscus repair. We should pay more attention on how to exert their regenerative properties and maintain effective directional differentiation. The question seems to be the applicability of MSCs for meniscus regeneration, rather than their availability in researches.

## 3 Approaches to MSC Utilization

**FIGURE 2:**
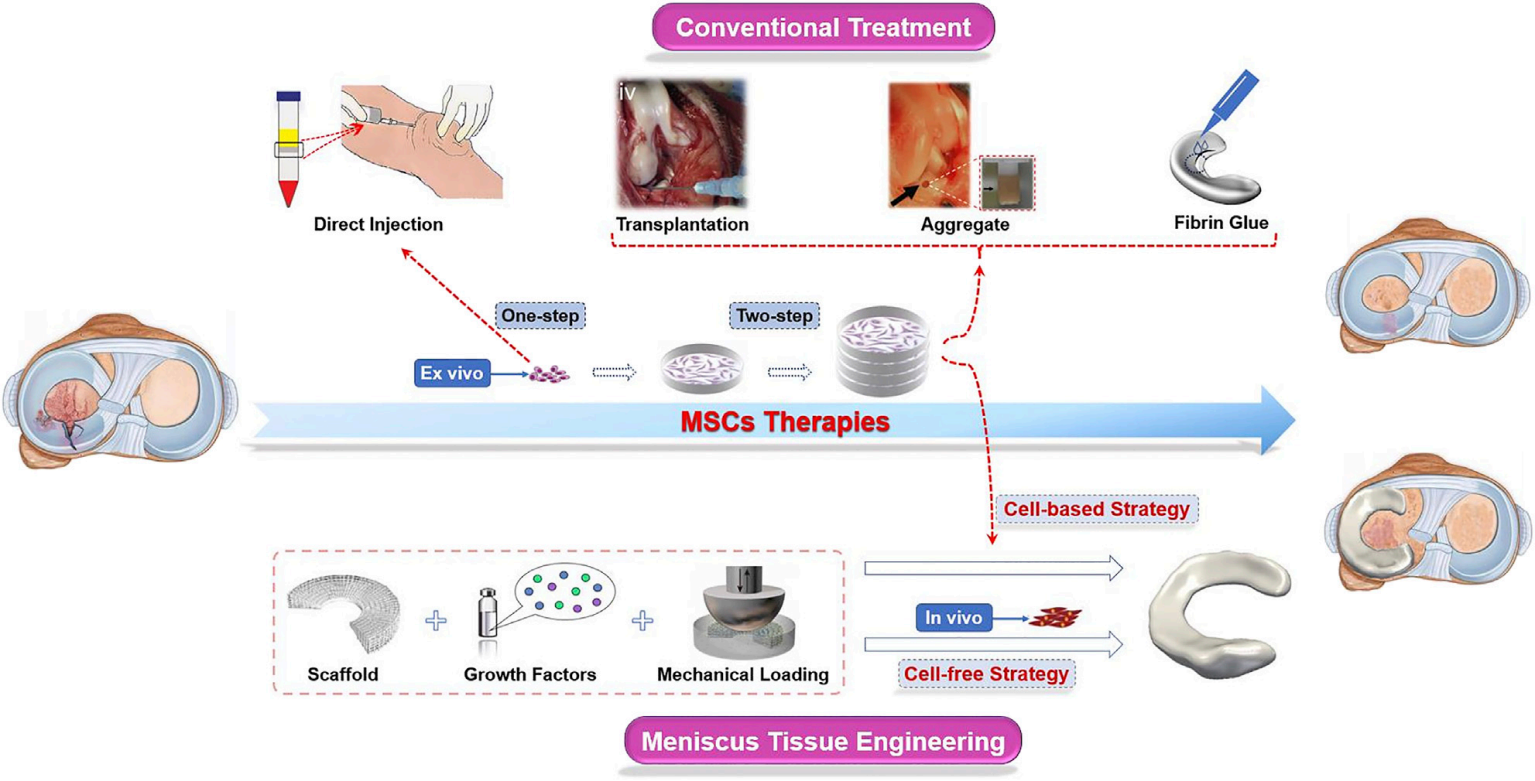
Schematic diagram of MSC therapies used in meniscus regeneration. In conventional approaches, MSCs are transplanted into the knee joints after separation (one-step) and/or culturing (two-step) *ex vivo*. Cells, scaffolds, and stimuli such as biochemical factors and biomechanical loading are indispensable components in the novel meniscus tissue engineering (MTE) strategy. Scaffolds are seeded with MSCs amplificated *ex vivo* before being implanted into the knee joints is a “cell-based” strategy, while the “cell-free” strategy is aimed at repairing the meniscus by recruiting endogenous MSCs. These images were reproduced with permission (Horie et al., 2012b; Nakagawa et al., 2015; Toratani et al., 2017a; Tarafder et al., 2018; Zhang et al., 2019).

There are roughly two ways to deliver MSCs into the impaired site: one is the direct injection of a suspension into the knee (one-step), and the other is first to preculture and expansion *in vitro* and then implant into the defect zone (two-step) (Anderson et al., 2014). Scaffold-free strategies, such as “high-density” cell aggregation or fibrin-based solutions, promoted histological meniscus healing in animal models, which are also promising repair techniques for meniscal tears (Toratani et al., 2017b; Marom et al., 2021). However, in large meniscal defects, MSCs usually exert only limited biological response in the process of meniscal regeneration for the lack of attachment site. Therefore, carriers and scaffolds are commonly used in the damaged site to provide a platform for cell attachment, proliferation, and differentiation (Tanaka et al., 2010). The technique of partially or totally replacement of damaged menisci with artificial engineered constructs is known as meniscus tissue engineering (MTE), a novel approach different from traditional MSC treatments (Figure 2). Generally, the one-step cell-based method is usually used to treat meniscus injuries by isolating and transplanting MSCs. The two-step approach requires preculture and expansion *in vitro*. The expanded cells from a variety of tissues could be transplanted into the damaged meniscus areas alone or carried by biomaterials (Table 1).

**TABLE 1:** Classification based on approaches of MSC therapies.

Approaches	Cell	Cell implant	Dosage of cells	Outcome	Animal model
Intra-articular injection	BMSC	One-step, once	2 ml/knee	Improved meniscal wound healing	Dog
		Two-step, once	5×10^6^ cells/knee	Adhered to the lesion, differentiated into meniscal cells directly, and promoted meniscal regeneration	Rat
	SMSC	Two-step, repetitive	5×10^7^ cells/knee	Defect filled with synovial tissue. Articular cartilage and subchondral bone were effectively preserved	Pig
Transplant to meniscal lesion sites	ADSC	Two-step, once	1×10^5^ cells/model	Meniscal healing was histologically increased when suture immediately	Rabbit
	SMSC	Two-step, once	2×10^7^ cells/model	Adhered to injury sites, differentiated into fibrochondrocytes, and enhanced meniscal regeneration and tensile strength	Rabbit, microminipig
Aggregate	ADSC	Two-step, once	1 cylindrical plug/model	Adhered to the defect and promoted histological meniscus healing	Rabbit
	SMSC	Two-step, once	500×10^6^ cells/aggregate, 1–50 per knee	Promoted meniscus regeneration and delayed progression of degeneration of articular cartilage	Primates, rat, pig
Fibrin glue	BMSCs	Two-step, once	1×10^6^ cells/model	MSCs in fibrin glue significantly produced an abundant ECM, increased total bonding, and enhanced meniscal healing, but the mechanical properties of the repair tissue decreased	Rabbit, rat, pig, horse
Tissue-derived materials	None	—	SIS scaffold without cells	Be conducive for cellular repopulation with host meniscal characteristics and be capable of supporting the complete healing of a large defect. But cartilage degeneration happened	Rabbit, goat, dog
	BMSC	Two-step, once	Unspecified silk fibroin scaffolds incubated in BMSC-rich well	Showed compatibility and feasibility of structure, and function in meniscal repair	Rabbit
	Coculture (MC + SMSC)	Two-step, once	0.9×10^6^ cells/construct	Enhanced cell survival and differentiation into chondrogenic phenotypes	Pig
ECM component-derived bioscaffolds	None	—	Acellular test	Improved the joint contact mechanics	*In vitro*
	MC	—	Unspecified	Be capable of MC attraction and matrix formation	*In vitro*
	SMSCs	Two-step, once	4.4×10^5^ SMSCs/model	Effectively promoted cellular infiltration, proliferation, survival, migration, and proliferation	Dog
	MMSC	Two-step, once	1–1.3×10^6^ cells/model	Increased cell proliferation and chondrogenic gene expression, and improved mechanical properties	Rabbit, dog
Synthetic polymeric scaffolds	None	—	PCL and HC (or PLLA and PGA) hybrid scaffolds without cell seeding	Histological investigation revealed tissue formation, cellular infiltration, and vascularization. Possessed biological and biomechanical functions for meniscal regeneration	Rabbit, sheep
	MC	Two-step, once	∼10^6^ cells/ml seeded onto PLDLA/PCL-T scaffolds	Increased the formation of fibrocartilaginous tissue, PEG increased Col II mRNA expression, and higher GAG production	Rabbit, sheep
	BMSC	Two-step, once	4–5×10^6^ cells/scaffold	Increased well-integrated fibrocartilaginous tissue regeneration and mechanical strength	Rabbit
Hydrogels	None	—	Artificial hydrogel without cell seeding	Improved the contact mechanics. Promising results at early times, but joint degeneration and implant failure 1 year later	Sheep, ovine
	MC	Two-step, once	5×10^7^ cells/ml gel, or 2×10^5^ cells/scaffold	Had good compatibility with MCs, growth factor increased the mechanical and biochemical properties. Promoted cell proliferation and fibrocartilaginous ECM production	*In vitro*
	BMSC	Two-step, once	3×10^7^ cells/ml gel	MSCs in meniscus ECM hydrogel enhanced tissue regeneration and protection from joint deterioration	Rat
	Coculture (MC + BMSC)	Two-step, once	0.5×10^5^ cells/ml gel	Increased meniscus-like ECM production	Equine

In this section, we summarized the main approaches of MSCs for meniscal repair. For the sake of description, we have artificially defined them as conventional strategies and novel approaches, and the latter mainly includes MTE (based on scaffold), while the rest (cell aggregate or fibrin-based solutions) are included in the former. In particular, direct injection or delivery *via* various scaffold media/materials are commonly used avenues to directional implantation for cell-based techniques. The cell-free strategy is based on the recruitment of endogenous MSCs.

### 3.1 Conventional Strategies

#### 3.1.1 Intra-Articular MSC Injection

The intra-articular injection was a commonly used method to treat meniscal lesions in early research works (Abdel-Hamid et al., 2005; Horie et al., 2012a; Hatsushika et al., 2014). 2 ml autologous bone marrow cells were separated by centrifugation and injected into the dog articular cavity (Abdel-Hamid et al., 2005). MSCs were also injected into knee joints by cell counting (2–6×10^6^ or 5×10^6^ in rat models (Horie et al., 2009; Horie et al., 2012a; Shen et al., 2014) and 5×10^7^ in pig models (Hatsushika et al., 2014)) rather than by volume. These cells were injected into the knees immediately after the skin incision closed (one injection only) or 2 weeks later (three times injection at 2-week intervals). The angiogenesis, chondrogenesis, immune cell infiltration, and collagen deposition of repaired menisci in the injected joints were much higher than those in the non-injected object. Needles were often used to deliver the cells directly transplanted to the targeted damaged sites (Horie et al., 2012b; Nakagawa et al., 2015). The knees were kept still for a few minutes after solution injection, then the sutures were tightened, and the capsule and skin were closed. Miguel et al. (Ruiz-Ibán et al., 2011) also reported meniscal healing by using ADSCs in the same way. These cells were suspended in the gel phase after *in vitro* culture and then instilled into the lesion followed by tightening the knots. The results manifested that the healing of the avascular meniscus was improved, especially in the acute phase. The expanded MMSCs between P1∼P3 were injected into rat meniscectomy models. The results verified the contribution on meniscal repair for the more neo-tissue formation and ECM deposition at an early stage after injection (Shen et al., 2014). Another method of cell transplantation is the aggregation of MSCs. The hanging drop culture method was used in cultivating SMSCs, which contained 2.5×10^5^ MSCs for each after culturing 3 days, and then several aggregates were placed in the meniscus (Kondo et al., 2017). ADSCs were cultured and aggregated into a spheroid structure and transplanted into the meniscal defect (Toratani et al., 2017a). These results showed that transplantation of aggregates could promote meniscus regeneration and delay the progression of degeneration of articular cartilage. What is more, it has been reported that transplantation of aggregates of SMSCs regenerated the meniscus more effectively than intra-articular injection with the same cell number used (Katagiri et al., 2013). In general, studies in direct injection of MSCs were still relatively limited, and there was no clear definition of the optimal number of cells to achieve the satisfying therapeutic effect. We proposed that the animal species may differ in their need of cell number; animals with larger joints generally require more cells. Although the aggregate contained more cells for its *ex vivo* culture, it does not seem to be a widespread method in meniscus repair studies.

One-step cell implantation was not widely used in the studies because of limited cell numbers. In fact, more research works had adopted the two-step method. The MSCs are usually implanted into the joints by cell suspension or aggregation. In order to enable MSCs to perform their biological function in a specific region, certain vehicles were usually used for transportation, such as liquid fibrin glue and specific shaped scaffold. The former helps MSCs gather and stick to the damaged area, and the latter provides a platform for cell attachment, and its shape serves as a template reference for the finally formed tissue.

#### 3.1.2 Fibrin Glue

Fibrin glue loaded with BMSCs has been reported to treat meniscal defects in rabbits. Healing of meniscus tissues had been observed 3 weeks after the operation, which showed better short-term results than those treated with acellular fibrin glue (Ishimura et al., 2000). The results were different from those reported in 1996, which indicated that the combination of autologous bone marrow cells and exogeneous fibrin clots would not enhance the meniscal healing (Port et al., 1996). The disparate results from these studies might be on account of the advancement in extraction and utilization of MSCs, and an improved property of fibrin glue. In subsequent studies, transplanted green fluorescent protein (GFP) cells carried by fibrin glue were detected in rats until 8 weeks. This study clearly demonstrated that allogenic MSCs embedded in fibrin glue could survive and proliferate in the meniscal defect in the avascular status, and promote the meniscal healing process (Izuta et al., 2005). BMSCs could sustain abilities of survival and differentiation when integrated with fibrin glue (Dutton et al., 2010), and the combined constructs implanted subcutaneously in nude mice showed significantly increased vascularization and tissue bonding (Ferris et al., 2012). What is more, a novel approach to treat avascular meniscus tears without exogenous stem cells has been reported (Tarafder et al., 2018). Endogenous SMSCs could be recruited, and fibrous matrix could be generated with the induction of growth factors loaded in fibrin glue.

### 3.2 Novel Approaches

**FIGURE 3:**
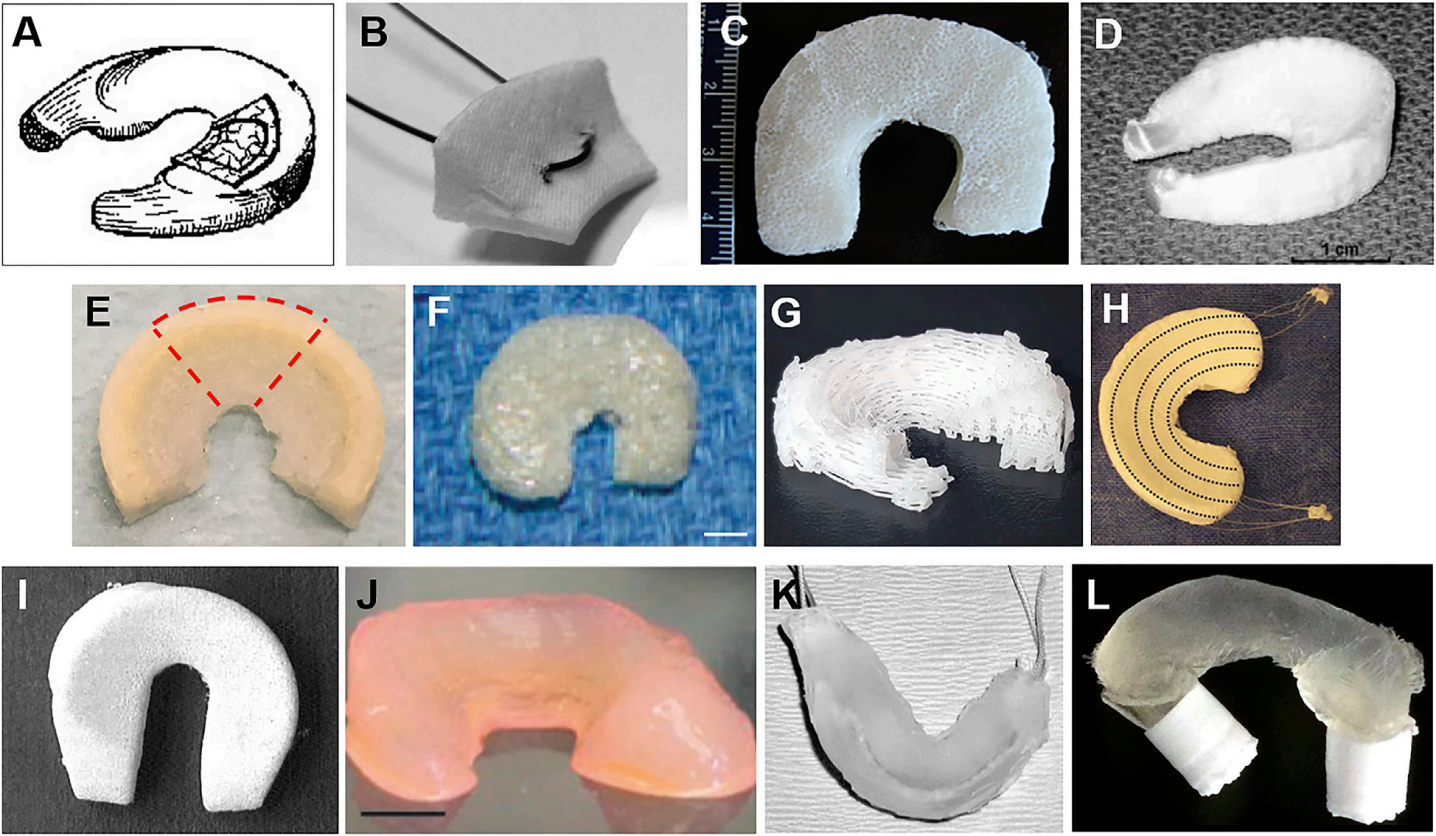
Different kinds of meniscus scaffolds. **(A)** The SIS meniscal implant (Cook et al., 2006) was used in a dog model. **(B)** The collagen meniscal implant (Gwinner et al., 2017) was sectioned radially, creating specimens of 15 mm width to measure its biomechanical properties. **(C)** Multiporous silk scaffold composing of three individual layers with different pore sizes and orientations in each layer (Mandal et al., 2011). **(D)** Fiber-weaved meniscus scaffold from bovine dermal collagen reinforced by a network of degradable tyrosine-derived polymer fibers (Balint et al., 2012). **(E)** The 3D-printed polymer network infusing with collagen–hyaluronic acid (Ghodbane et al., 2019b). **(F)** Meniscus-shaped PGA mesh scaffold of a rabbit model (scale bar: 4 mm) (Kang et al., 2006). **(G)** A 3D-printed PCL meniscus scaffolds with a biomimetic fiber architecture (Szojka et al., 2017). **(H)** A porous scaffold composited of PCL and HYAFF^®^ and augmented with circumferential PLA fibers (Chiari et al., 2006). **(I)** A meniscus scaffold fabricated by PCLPU for a dog model (Welsing et al., 2008). **(J)** Anatomically shaped alginate meniscus (scale bar: 5 mm) (Puetzer et al., 2013). **(K)** Hydrogel meniscal construct with sutures woven through the anterior and posterior horn used in a sheep model (Kelly et al., 2007). **(L)** Thermoplastic elastomer (TPE) hydrogel with tabs swollen into PLA cylinders (Fischenich et al., 2018).

Fibrin glue enhances the reconnection level in the torn meniscus, and the MSCs strengthen the neo-tissue formation and ECM deposition. For meniscus defect, cells lose the substrate to adhere and further differentiate, leading to less effective tissue regeneration. Artificial engineered scaffolds might ideally compensate for this. As an integral part of tissue engineering, scaffolds could partially or completely replace the impaired or degenerated menisci. The goal of MTE is to finally generate constructs that mimic native gradient menisci. Scaffolds could be generally categorized into four classes based on their compositions (Zhang et al., 2015): tissue-derived materials, ECM component-derived bio scaffolds, synthetic porous polymeric scaffolds, and hydrogels (Figure 3).

#### 3.2.1 Tissue-Derived Materials

Small intestinal submucosa (SIS), an early published material type, was commonly used in soft-tissue repair after the removal of all cellular and nuclear materials. Its mechanical and biological properties would be beneficial to cell infiltration, matrix formation, and neo-tissue remodeling after scaffold biodegradation (Gastel et al., 2001; Bradley et al., 2007). It was reported (Tan et al., 2010) that SIS scaffolds consisted of 80–90% collagen with oriented fibers, containing GAGs and soluble factors. SIS scaffold implantation resulted in more meniscus-like tissue production than meniscectomy, and seeding cells on SIS seemed a promising approach to meniscus repair (Cook et al., 2006). Though this material has many advantages, like high bioactivity, it has not gained enough attention at present due to several drawbacks: limited sources and insufficient mechanical strength. Silk fibroin is another material derived from tissue with outstanding biocompatibility and mechanical properties. Silk fibroin solution (Mandal et al., 2011) was prepared from *Bombyx mori* silkworm cocoons and then further fabricated into a scaffold. The cell-seeded silk constructs confirmed the deposition of sGAG and Col I and II by histological and immunohistochemical assessments. The histocompatibility and feasibility of structure and function, as well as controlled degradability, were also confirmed by various studies (Wang et al., 2008; Ying et al., 2018). Combined silk scaffolds (Liu et al., 2015) and composited scaffolds (Shi et al., 2017) were successfully explored and used for tissue regeneration in combination with MSCs, and they were fabricated by forming microporous silk sponges in the knitted silk or designed by integrating silk fibroin with gelatin. These findings demonstrated that the tissue-derived scaffolds have their own advantages, especially in biocompatibility. Nevertheless, few research works focused on this kind of scaffold, probably on account of their limited sources.

#### 3.2.2 ECM Component-Derived Bio Scaffolds

ECM plays a vital role in maintaining the biological and biomechanical properties of native menisci. Similar to tissue-derived scaffolds, ECM-derived biomaterials are also non-cellular tissues that provide platforms for cell attachment and regulate cell differentiation, matrix generation, as well as tissue morphogenesis and homeostasis (FA and Cook, 2017). Theoretically, the tissue-derived ECM biomaterials can serve as a substrate that is close to the native state and contribute to cell attachment and proliferation. Collagen is the dominant protein in the ECM, and collagen meniscus implant (CMI) is the only Food and Drug Administration (FDA)-approved partial meniscus substitution that serves as a biodegradable template for cell ingrowth (Gwinner et al., 2017). Hyaluronan could play an important role in collagen remodeling after meniscus injury (Sonoda et al., 2000). Derived from natural sources, collagen-derived scaffolds might possess favorable biocompatibility and biodegradability for cell seeding (Kremer et al., 2017). Collagen and hyaluronic acid composited scaffolds replicate the key structure and load-distribution properties of the native meniscus, which could approximately restore the joints’ normal axial compression and circumferential tensile stress (Ghodbane et al., 2019a). Histological evidence, ECM deposition, and immunohistochemical staining born out that scaffolds were effectively involved in meniscus tissue reconstruction (Merriam et al., 2015). ECM-derived components are essential subsets of biomimetic scaffolds, which hold excellent properties for cell attachment and proliferation, as well as biofunction restoration. It is worth noticing that the scaffolds were reinforced by polymer fiber fabricated by the 3D technique in recent research works. The voids of the 3D-printed polymer are beneficial for the infiltrating of MSCs, and the composited biomaterials with different mechanical properties provide substrates for cellar anisotropic differentiation.

#### 3.2.3 Synthetic Porous Polymeric Scaffolds

Multiple sources of materials were reported in the research works of synthetic bioscaffolds up to now, such as poly (L-lactide) (PLLA), poly (p-dioxanone) (PPD), polyglycolide (PGA), poly (lactide-co-glycolide) (PLGA), poly (ε-caprolactone) (PCL), and polyurea–poly (L-lactide) (PU–PLLA) (Koller et al., 2012; Esposito et al., 2013; Rongen et al., 2014; Murakami et al., 2017; Shimomura et al., 2017; Zhang et al., 2017; Koch et al., 2018). Electrospinning techniques and 3D printing technology are commonly used methods to obtain patient-specific constructs, aiming at narrowing the morphological differences between the artificial material and native tissue (López-Calzada et al., 2016; Szojka et al., 2017). The porous structure provided avenues for cell infiltration and ECM deposition, and facilitated the neo-tissue formation. Biomimetic porous polymeric scaffolds fabricated by the 3D printing technology have been receiving growing attention in MTE research in recent years (Williams et al., 2018). Different origins of materials, construct architectures, and manufacturing methods have been developed to fabricate scaffolds (Szojka et al., 2017). In general, the circumferential and internal fiber orientation was designed to mimic the skeleton of the native menisci. Mechanical stimulation and biochemical factors were selectively added to the material or the construct to enable seed cells to differentiate into the targeted mature type and construct anatomically zone-specific menisci (Zhang et al., 2015). Both cellular and acellular synthetic scaffolds were implanted into the meniscal defects. Encouragingly, both of them had finally generated anisotropic menisci, making a great progress in MTE (Lee et al., 2014). Although there are still some disadvantages like lack of bioactivity and hydrophilic properties, these materials still play an important role and hold new promising prospects in meniscal regeneration.

#### 3.2.4 Hydrogel Scaffolds

Hydrogels, also known as hydrophilic gels or sometimes colloidal gels, are polymer networks based on cross-linked hydrophilic polymers with water as the dispersion medium (Ahmed, 2015). This material could be made from a wide range of natural and synthetic polymers, and classified into chemical and physical categories according to their cross-linking mechanism (Zhu and Marchant, 2011). The chemical cross-linked structures are formed by covalent or ionic bonds with closely aligned and permanent junctions, while the other is weakly interacted by physical twining or connected by hydrogen bonds (Slaughter et al., 2009). Natural polymer-derived hydrogels, such as fibrin, alginate, gelatin, and collagen, are liable to mimic the native menisci with outstanding biocompatibility and biodegradability, but the weak mechanical properties make this material expose to the risk of breakage, while the lack of adequate biological activities is the biggest drawback for synthetic hydrogel (Brandl et al., 2007; Kelly et al., 2007; Puetzer et al., 2013). The synthetic and natural hybrid hydrogels (i.e., the combination of these two polymers) have been employed to control the scaffolds’ matrix architecture, which could simultaneously affect the cellular response (Anjum et al., 2016).

Hydrogel scaffold implantation could partially restore contact area and pressure distribution of the knee, especially in the lateral compartment (Fischenich et al., 2018). Compared with 3D PCL scaffolds with stronger mechanics, the hydrogel scaffolds were more conducive to the production of meniscus ECM (Bahcecioglu et al., 2019b). Moreover, the sources of hydrogel might exert influences on the heterogeneous differentiation of the MSCs. Recently, a PCL/hydrogel composite biomaterial has been explored (Bahcecioglu et al., 2019a). Bahcecioglu G et al. impregnated agarose (Ag) and methacrylated gelatin hydrogels into PCL scaffolds in the inner and outer regions, respectively. The constructs generated cartilage-like tissue at the inner zone and fibrocartilage-like tissue at the outer zone, thus showing a new promising approach to engineer an anisotropic substitution. Hydrogels’ networks can also provide platforms for the incorporation of bioactive molecules or MSCs according to the need of the research (Koh et al., 2017; Yuan et al., 2017). This controlled delivery system could be designed because the physicochemical properties of the hydrogels are sensitive to pH, temperature, and chemical reaction. The adjustable release of these substances in time and space might affect the cellular differentiation and tissue remodeling. Overall, hydrogels could be used as not only carriers but also scaffolds. However, what should be borne in mind is that it is necessary to balance the relationship between mechanical strength and biological properties. To solve this problem, the combination of materials from different sources might provide a feasible approach. Several injectable hydrogels were used as a carrier to deliver MSCs, most of which are based on a natural hydrogel and reinforced with synthetic materials (Zhong et al., 2020; An et al., 2021).

## 4 Conclusion and Prospect

Taken together, current research works are more focused on scaffolds seeded with MSCs. MTE is an ever-growing research field with emerging strategies that aim to restore and improve meniscal function. Various engineered scaffolds have been successfully fabricated with bionics of morphology and structure at present, which has made a great progress in meniscus regeneration. Although the cell–scaffold composite shows excellent healing potential, the cell-based two-step strategy has its drawbacks including cell contamination in the process of cell expansion *in vitro*. The cell-free method with one-step scaffold implantation has been getting more and more attention. This technique effectively avoids the complications in the procedure of cell culturing *in vitro*. This technique might provide greater expectation for the research works of meniscus regeneration in the future if enough MSCs could be collected and differentiated into fibrochondrocytes. How to get these materials more involved in the biological reaction play their regulatory role in cell-directed differentiation and ultimately form a substitute with heterogeneity in composition, and biomechanical properties need further investigation.

Meniscus lesion is a common knee injury and usually results in degenerative changes of the joints. It is hard to repair spontaneously once injured or defected, especially for the white region, which refers to the region with poor vascular supply and low cellularity. MSCs are widely used in meniscus regeneration because of their abundant sources, availability, and outstanding differentiation capability. Conventional treatments with MSCs include direct application, articular injection, and transplantation of aggregates. MTE is a burgeoning approach for meniscus regeneration, which is composed of several elements such as seeding cells and various scaffolds. MSCs are the basis for matrix formation, while scaffolds are essentially engineered replacements that provide mechanical support, promote cell adhesion and growth, and guide three-dimensional tissue formation. Despite that MTE has made significant progress in recent years, research on the engineered meniscus regeneration using MSC is still in the laboratory or animal exploration stage. How to realize the clinical transformation of regenerative medicine and bring the benefits to more patients with meniscus injury is the ultimate goal of our research. Accordingly, more valuable research works are needed to explore anisotropic meniscus substitution in line with clinical demand.

## Data Availability

The original contributions presented in the study are included in the article/Supplementary Material, and further inquiries can be directed to the corresponding author.
